# Urinary *N*-methylnicotinamide and β-aminoisobutyric acid predict catch-up growth in undernourished Brazilian children

**DOI:** 10.1038/srep19780

**Published:** 2016-01-27

**Authors:** Jordi Mayneris-Perxachs, Aldo A.M. Lima, Richard L. Guerrant, Álvaro M. Leite, Alessandra F. Moura, Noélia L. Lima, Alberto M. Soares, Alexandre Havt, Sean R. Moore, Relana Pinkerton, Jonathan R. Swann

**Affiliations:** 1 Division of Computational and Systems Medicine, Department of Surgery and Cancer, Imperial College London, UK; 2 Department of Physiology and Pharmacology & INCT-Biomedicine, Faculty of Medicine, Federal Unversity of Ceará, Fortaleza, Ceará, Brazil; 3 UVA Center for Global Health, Division of Infectious Diseases and International Health, University of Virginia, School of Medicine, Charlottesville VA, US; 4 Institute for the Promotion of Nutrition and Human Development & Department of Child and Maternal Health, Faculty of Medicine, Federal University of Ceará, Fortaleza, Ceará, Brazil; 5 Division of Gastroenterology, Hepatology, & Nutrition, Center for Global Health, Department of Pediatrics, Cincinnati Children’s Hospital Medical, University of Cincinnati, USA

## Abstract

Enteric infections, enteropathy and undernutrition in early childhood are preventable risk factors for child deaths, impaired neurodevelopment, and later life metabolic diseases. However, the mechanisms linking these exposures and outcomes remain to be elucidated, as do biomarkers for identifying children at risk. By examining the urinary metabolic phenotypes of nourished and undernourished children participating in a case-control study in Semi-Arid Brazil, we identified key differences with potential relevance to mechanisms, biomarkers and outcomes. Undernutrition was found to perturb several biochemical pathways, including choline and tryptophan metabolism, while also increasing the proteolytic activity of the gut microbiome. Furthermore, a metabolic adaptation was observed in the undernourished children to reduce energy expenditure, reflected by increased *N*-methylnicotinamide and reduced β-aminoisobutyric acid excretion. Interestingly, accelerated catch-up growth was observed in those undernourished children displaying a more robust metabolic adaptation several months earlier. Hence, urinary *N*-methylnicotinamide and β-aminoisobutyric acid represent promising biomarkers for predicting short-term growth outcomes in undernourished children and for identifying children destined for further growth shortfalls. These findings have important implications for understanding contributors to long-term sequelae of early undernutrition, including cognitive, growth, and metabolic functions.

Childhood undernutrition is a major global health problem with both acute and long-term consequences. One in five children in the developing world is undernourished and malnutrition is an underlying cause of more than half (53%) of all deaths worldwide in children aged younger than five years^1^,^2^. In addition to its role in childhood deaths, undernutrition in early life leads to poor ponderal and linear growth, which reflects the combined effects of both acute (wasting) and chronic undernutrition (stunting). Nutritional interventions have reduced mortality in undernourished children but the long-term consequences for children who survive remains a major challenge. Two decades of accumulating evidence link low birth weight to an increased risk for both cardiovascular disease (CVD) and type 2 diabetes. More recent studies indicate that early childhood stunting is associated with cognitive impairment and increased risk of obesity and CVD in later life^3^. However, the mechanisms underlying these long-term outcomes have not yet been defined.

One variable that may contribute to these outcomes is the collection of microbes residing in the gastrointestinal tract. These gut microbiota have been implicated in gut inflammation, obesity, CVD risk and through the gut-brain axis have been shown to influence brain development^4^,^5^,^6^. By providing the host with a range of extra-genomic metabolic functionality these intestinal microbes extend the biochemical flexibility of the host and hold a significant role in human nutrition. Increasing evidence is emerging to show that early-life malnutrition retards the maturation of the gut microbiota and host acquisition of these metabolic capabilities^7^,^8^,^9^. It is plausible that modulations in gut microbial community assembly could contribute the outcomes associated with early-life nutrition.

Metabolic phenotyping involves the measurement of global sets of metabolites within a biological system and their variation in response to stimuli. Metabolic profiles are the product of genetic and environmental (diet, lifestyle, gut microbial activity) contributions and studying these profiles enables the overall metabolic status of this multi-factorial metabolic system to be assessed. These signatures contain information reflecting healthy physiological processes and pathological events and can be studied to identify metabolic pathways underlying disease risk and for discovering molecular diagnostic and predictive biomarkers. Large-scale metabolic phenotyping (molecular epidemiology) for biomarker discovery has been previously applied to identify unique metabolic signatures of nutrition and aging, and also disease states including autism, prenatal disorders, type 2 diabetes, stress, depressive disorders, hypertension, and CVD^10^,^11^,^12^,^13^,^14^,^15^,^16^,^17^,^18^,^19^.

In the present work, a metabolic phenotyping approach was applied to characterize the biochemical modulation induced by undernutrition to understand the biomolecular mechanisms underlying undernutrition associated growth and potential cognitive impairment. We have applied a ^1^H nuclear magnetic resonance (NMR) spectroscopy-based metabolic profiling approach to study the urinary metabolites of children enrolled in a case-control study in Northeast Brazil. This study was supported as a part of the ‘Etiology, Risk Factors and Interactions of Enteric Infections and Malnutrition and the Consequences for Child Health and Development’ (MAL-ED) study. Urinary metabolic profiles were studied to elucidate potential biomarkers of undernutrition and to predict subsequent “catch-up” growth in these children. We report here a potential mechanistic role for altered choline and tryptophan metabolism in the effects associated with undernutrition and a potential role for increased nicotinamide *N*-methyltransferase (NNMT) in catch-up growth with *N*-methylnicotinamide serving as a biomarker for this adaptation.

## Results

### Metabolic signature of undernutrition in the urinary metabolome

**Figure 1:**
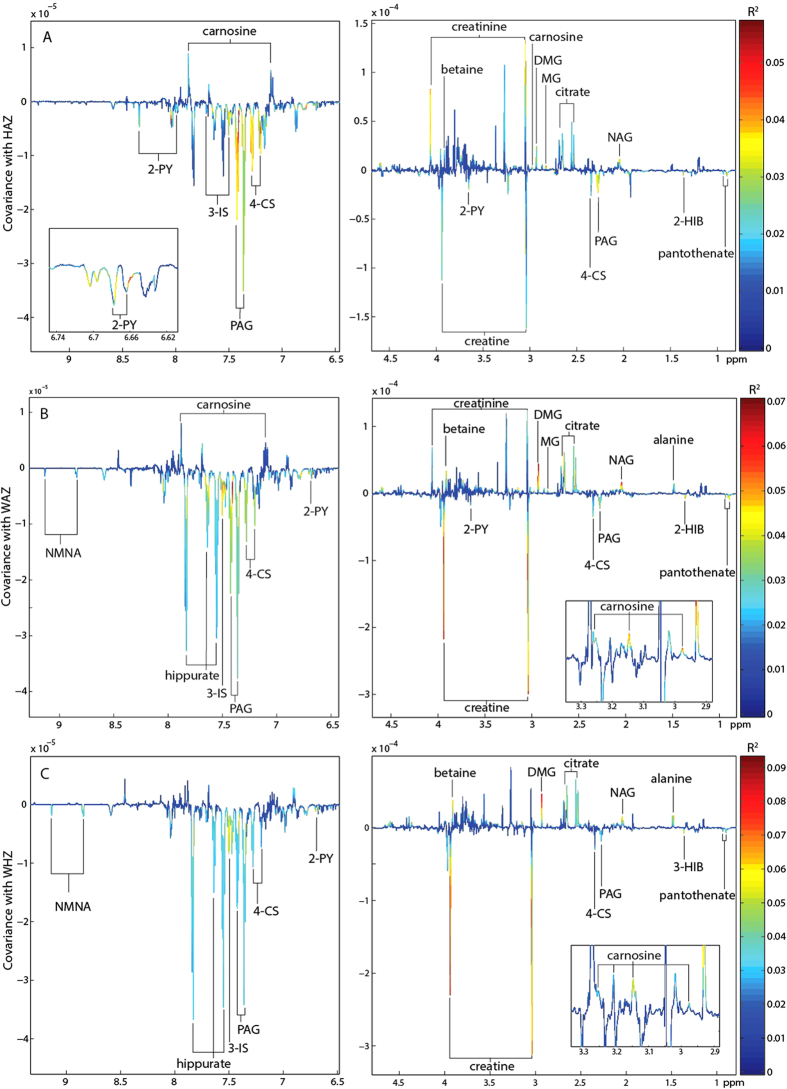
Orthogonal projection to latent structures (OPLS) model identifying metabolic associations with measures of undernutrition. Coefficients plot extracted from the OPLS model constructed from the urinary ^1^H NMR profiles and corresponding (**A**) height-for-age *z* (HAZ)-scores (**B**) weight-for-age *z* (WAZ)-scores and (**C**) weight-for-height *z* (WHZ)-scores. 2HIB, 2-hydroxyisobutyric acid; 2-PY, *N-*methyl-2-pyridone-5-carboxamide; 3-IS, 3-indoxyl sulfate; 4-CS, 4-cresyl sulfate; DMA, dimethylamine; DMG, dimethylglycine; MG, methylguanidine; NAG, *N* acetylglycoprotein; NMNA, *N*-methylnicotinic acid; PAG, phenylacetylglutamine.

**Figure 2:**
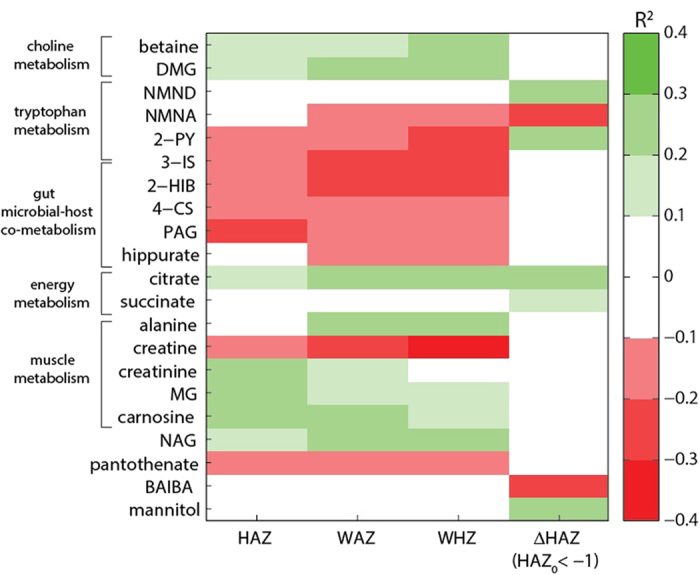
Summary of the metabolites associated with the OPLS models given by the correlation coefficient (R) with the response variable. HAZ, height-for-age *z*-score; WAZ, weight-for-age *z*-score; WHZ, weight-for-height *z*-score; ΔHAZ, change in height-for-age *z*-score over two to five months. 2HIB, 2-hydroxyisobutyric acid; 2-PY, *N-*methyl-2-pyridone-5-carboxamide; 3-IS, 3-indoxyl-sulfate; 4-CS, 4-cresyl sulfate; BAIBA, β-aminoisobutyric acid; DMA, dimethylamine; DMG, dimethylglycine; MG, methylguanidine; NAG, *N* acetylglycoprotein; NMNA, *N*-methylnicotinic acid; NMND, *N*-methylnicotinamide; PAG, phenylacetylglutamine.

Orthogonal projections to latent structures (OPLS) models with one predictive component and no orthogonal components were calculated to identify urinary metabolites associated with undernutrition in children from Northeastern Brazil (*n* = 326; 6.2–25.9 months old; 161 male and 165 female). Descriptive information on the children included in these models can be found in the Supplementary Information (Tables S1 and S2). Unit-variance-scaled NMR spectra acquired from urine samples served as the descriptor (X) variables and metrics of nutritional status including height-for-age *z* scores (HAZ), weight-for-age *z* scores (WAZ), and weight-for-height *z* scores (WHZ) were used individually as the response (Y) variable. Valid OPLS models were returned for all metrics analyzed identifying metabolic variation associated with nutritional status (Table 1). Those metabolites associated with stunting (inversely associated with HAZ; where HAZ < −2 indicates moderate stunting) are shown in the coefficients plot extracted from the respective OPLS model (Fig. 1A). Here, stunting was positively associated with creatine, glycerophosphocholine (GPC), *N-*methyl-2-pyridone-5-carboxamide (2-PY), and pantothenate excretion. In addition, a positive correlation was observed with metabolites arising from gut bacterial-host co-metabolism. This included 2-hydroxyisobutyrate (2-HIB), phenylacetylglutamine (PAG), 4-cresyl sulfate (4-CS), and 3-indoxyl sulfate (3-IS). In contrast, stunting was negatively associated with the excretion of *N*-acetyl glycoproteins (NAG), citrate, methylguanidine (MG), dimethylglycine (DMG), carnosine, creatinine, betaine, and unknown resonances observed at δ 3.95 and 4.60. Similar observations were found for the models considering wasting (WHZ) and underweight (WAZ). In addition, WAZ and WHZ were also found to positively correlate with *N*-methyl-nicotinic acid (NMNA) and hippurate excretion and negatively correlate with alanine excretion (Fig. 1B,C). The correlation coefficients indicating the association of these metabolites with the various metrics of undernutrition are summarized in Fig. 2. Spectral peak integrals have been calculated for the metabolites identified to change with relation to these measures of nutritional status and are presented in the Supplementary Information Table S3.

### Urinary *N*-methylnicotinamide (NMND) predicts catch-up growth (ΔHAZ)

**Figure 3:**
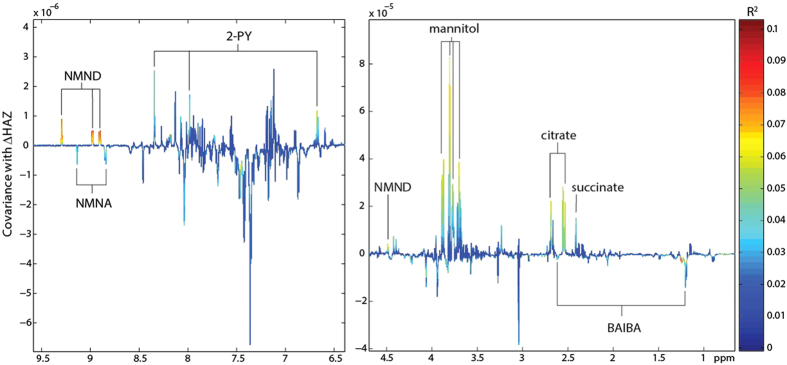
OPLS coefficients plot identifying metabolic predictors of growth in stunted children or those at risk of stunting. Baseline urinary metabolic profiles of children with a baseline HAZ <−1 correlated with ΔHAZ measured two to five months later. 2-PY, *N-*methyl-2-pyridone-5-carboxamide; BAIBA, β-aminoisobutyric acid; NMNA, *N*-methylnicotinic acid; NMND, *N*-methylnicotinamide.

Measures of the change in HAZ (ΔHAZ) from baseline to 2–5 month follow-up were available for 252 children whose urinary metabolic phenotypes were defined at baseline. Missing data analyses were performed and no significant differences were observed in gender (*p* = 0.52), age (*p* = 0.26) or HAZ status (*p* = 0.49) at baseline for children with or without follow-up anthropometry. OPLS models were constructed on baseline urinary metabolic phenotypes to elucidate metabolic information associated with subsequent growth 2–5 months later (ΔHAZ). No significant metabolic associations were observed with ΔHAZ when all children were modeled. However, stratifying the sample set to examine metabolic correlates with ΔHAZ in stunted children or those at risk of stunting (HAZ < −1 (more than one standard deviation below the population median); *n* = 173) returned a significant OPLS model (ΔHAZ range: −0.92–1.39; Fig. 3; metabolic associations (correlation coefficients) are summarized in Fig. 2). This model identified a positive relationship between the excretion of *N*-methylnicotinamide (NMND), 2-PY, succinate and citrate at baseline and growth (ΔHAZ) measured 2 to 5 months later. The baseline excretion of β-aminoisobutyric acid and *N*-methylnicotinic acid were also negatively correlated with ΔHAZ in these children. In addition, the excretion of mannitol, provided as part of the lactulose-mannitol test to assess small bowel surface area, was also positively associated with growth. Further refinement of the sample set to look at metabolic associations with ΔHAZ in stunted children (baseline HAZ < −2; *n* = 108; ΔHAZ range: −0.92–1.39) also returned a significant model (see Supplementary Fig. S1 online). The metabolic alterations observed with this model were consistent with those identified in the earlier model (baseline HAZ < −1). No association was identified between baseline metabolic signatures and ΔHAZ in the non-stunted (HAZ > −1; *n* = 82) children.

## Discussion

Combining field studies of undernutrition with a ^1^H NMR spectroscopy-based metabolic profiling approach we obtained a deeper understanding of the metabolic pathways altered in the setting of early childhood growth stunting and, in turn, identified potential biochemical mechanisms underlying undernutrition associated growth and cognitive impairment. In this study similar metabolic alterations were found across all measures of stunting (HAZ), underweight (WAZ) and wasting (WHZ) with the exception of NMNA and alanine, which were uniquely associated with measures of underweight and wasting, and creatinine which was unique to stunting and underweight. This is consistent with the coexistence of these anthropometric failures in the undernourished children from this cohort.

**Figure 4:**
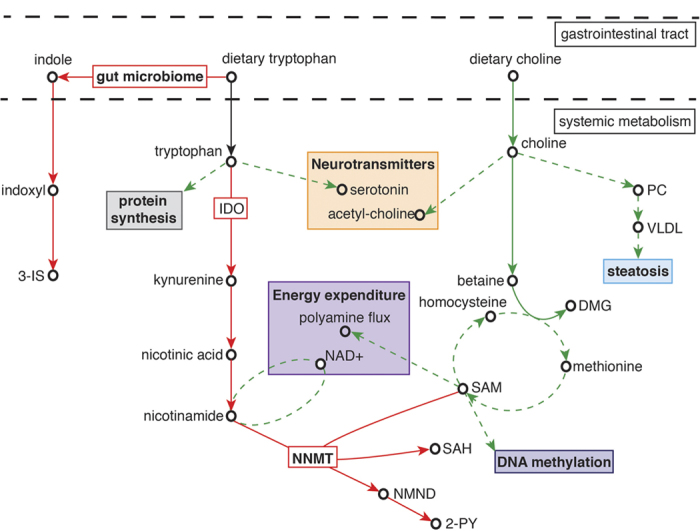
Metabolic pathways modulated by undernutrition. Red solid arrows indicate pathways increased with undernutrition (negatively associated with HAZ, WAZ, WHZ) and green solid arrows indicate pathways decreased with undernutrition (positively associated with HAZ, WAZ, WHZ). Dashed green arrows indicate pathways and biological functions hypothesized to decrease as a result of these metabolic alterations. 3-IS, 3-indoxyl-sulfate; DMG, dimethylglycine; IDO, indoleamine 2,3-dioxygenase; NAD, nicotinamide adenine dinucleotide; NMND, *N*-methylnicotinamide; NNMT, nicotinamide *N*-methyltransferase; PC, phosphatidylcholine; SAH, *S-*adenosyl-L-homocysteine; SAMe, *S*-adenosyl-L-methionine; VLDL; very-low-density lipoproteins.

Undernourished children were found to excrete lower amounts of betaine and dimethylglycine (DMG) compared to the nourished controls, indicating perturbations in choline metabolism (Fig. 4). Dietary choline is rapidly absorbed in the small intestine and catabolized to betaine and then DMG. Reduced excretion of these metabolites in the undernourished children indicates a lower availability of choline (via reduced intake or absorption) compared to nourished controls. Choline can be obtained from many dietary sources and the human infant typically consumes a choline-rich diet, with breast milk containing ~1.5 mmol/L choline and choline esters^20^. In this study no significant association was identified between breast-feeding and stunting (data not shown), although those children exposed to mixed breast-feeding practices did excrete more betaine and DMG compared to those not receiving breast milk (see Supplementary Fig. S2 online). However, when considering all study children simultaneously, stunting was found to negatively correlate with betaine and DMG excretion, which are surrogate markers of choline bioavailability, irrespective of breast milk exposure.

Choline and betaine are major sources of methyl groups used to generate the body’s primary methylating agent, *S*-adenosylmethionine (SAMe). Betaine is converted to DMG as it methylates homocysteine to methionine, which is subsequently used to generate SAMe. Lower excretion of betaine and DMG by undernourished cases is consistent with reduced flux through the betaine pathway to produce DMG and homocysteine. This has potential downstream consequences for SAMe abundance and others have demonstrated a strong positive correlation between plasma choline and plasma SAMe^21^.

SAMe serves as the sole methyl donor in a multitude of cellular methylation reactions including epigenetic processes such as DNA and histone methylation. Diet related modulation of DNA methylation has been previously shown in animal and human studies (reviewed in McKay *et al.* 2011^22^). For example, dietary choline deficiency results in DNA hypomethylation of mouse brains^23^. During early-life epigenetic regulation is dynamic and the epigenome has a developmental plasticity that is susceptible to dietary modulation. Alterations to these developing epigenetic marks have potential to modify gene expression and cellular function. Evidence is emerging that critical windows may exist to allow diet-associated variations in epigenetic programming to exert strong long-term impacts on health^24^,^25^. This was demonstrated in a rodent study where sustained methyl-group deficiency resulted in a reduced growth rate and elevated hepatic fat content^26^, outcomes attenuated by supplemental feeding of choline and methionine^27^. Exposure to a nutritionally limited environment in early-life is hypothesized to result in the fine-tuning of the epigenome to prime individuals towards metabolic “thrift”. Such early-life programming could compromise individuals in later life when this thrifty phenotype experiences an energy-rich environment. Consistently, deficiencies in both choline and betaine have been suggested to produce epigenetic changes in genes linked to atherosclerosis^28^ and brain development^23^,^29^.

One advantage of metabolic profiling is the ability to capture the influence of the gut microbiota on the metabolic status of the host. Increased excretion of gut bacterial-host co-metabolites derived from amino acid metabolism in the undernourished cases implies a functional modulation in the gut microbiome towards protein breakdown. This includes the excretion of phenylacetylglutamine (PAG), 4-cresyl sulfate (4-CS), and 3-indoxyl-sulfate (3-IS) derived from phenylalanine, tyrosine and tryptophan, respectively. Here, phenylalanine is converted to phenylacetate by the colonic bacteria and is subsequently conjugated with glutamine in the liver and the gut mucosa to form PAG. 3-IS and 4-CS are the products of the hepatic sulfation of indole and 4-cresol, which are end-products of the bacterial metabolism of tryptophan and tyrosine in the colon, respectively. Greater proteolytic activity of the undernourished microbiome is further supported by the increased excretion of 2-hydroxyisobutyrate, a bacterial degradation product of dietary proteins, in the undernourished children compared to the better nourished, control children^30^. Given that dietary intake is the main source of substrate for the gut microbiota it is unsurprising that undernutrition would imprint on microbiomic activity. Previous studies have shown gut microbial variation associated with kwashiorkor and with less severe forms of malnutrition^8^,^9^. A recent study in Indian children found that carbohydrate-active enzyme (CAZymes) families in the gut microbiome were positively associated with the nutritional status of the children^7^, consistent with our findings identifying a proteolytic enterotype associated with undernutrition. This may have consequences for the host given that metabolites generated from carbohydrate fermentation are generally considered beneficial for the host while those arising from protein fermentation tend to be more toxic^31^.

Greater urinary excretion of 3-IS and *N*-methyl-2-pyridone-5-carboxamide (2-PY) by the undernourished children suggests a dysregulation of tryptophan metabolism associated with undernutrition. There are three major metabolic routes for tryptophan degradation: the kynurenine, serotonin, and the indolic pathway. The kynurenine pathway is the major (>95%) catabolic route for tryptophan, resulting in the production of nicotinic acid, its amine form nicotinamide and several neuroactive intermediates (kynurenic acid, quinolinic acid, picolinic acid), whereas 1–2% of dietary tryptophan is used for the synthesis of the neurotransmitter serotonin. Approximately 4–6% of tryptophan undergoes bacterial degradation in the gut to produce indole, which is subsequently metabolized in the host to indoxyl and then to 3-IS or indoxyl-β-D glucuronide. In this study, undernutrition was positively associated with greater excretion of 3-IS, *N*-methylnicotinic acid, and 2-PY, a metabolite of nicotinamide, showing a greater metabolism of tryptophan through the bacterial-mediated indolic pathway and the endogenous kynurenine pathway with undernutrition (Fig. 4).

Tryptophan is an essential amino acid and its use in a wide variety of physiological functions result in a relatively low abundance in the body. Since tryptophan cannot be synthesized a large amount of tryptophan is used in protein synthesis and is essential for growth. In pigs, a moderate deficiency in tryptophan has been shown to significantly reduce growth rate^32^. Enhanced metabolism of tryptophan in the undernourished children through these two major pathways could reduce the bioavailability of tryptophan with potential implications for growth and serotonin biosynthesis.

Activation of the kynurenine pathway may also be driven by immune responses. Two enzymes are capable of metabolizing tryptophan to kynurenine. Tryptophan 2,3-dioxygenase (TDO) is primarily expressed in the liver and is considered a protective mechanism to prevent toxic amounts of tryptophan accumulating in plasma and tissues. Indoleamine 2,3-dioxygenase (IDO) is an enzyme present in several extra-hepatic tissues and its expression is increased in response to infection and inflammation^33^,^34^. One driver of systemic inflammation is increased intestinal permeability or a “leaky gut” frequently observed with undernutrition and persistent infection. This IDO-mediated activation of the kynurenine pathway depletes tryptophan from the local environment and deprives T-cells of tryptophan needed for activation and proliferation upon encountering potentially deleterious microbial antigens. In addition, downstream metabolites of the kynurenine pathway actively suppress the proliferation of activated T cells^35^. Therefore, inflammation-induced IDO activation down-regulates the initial immune response, leading to “secondary” immunosuppression. This is consistent with the lower excretion of *N*-acetylglycoprotein and carnosine in undernourished compared to control children. *N*-acetylglycoproteins are frequently associated with inflammation-induced acute phase proteins and several studies have demonstrated that carnosine has strong and specific antioxidant properties and the ability to promote wound healing^36^. In this context, carnosine scavenges reactive oxygen species generated by cells during an acute inflammatory response and enhances the process of wound healing by stimulating effusion at the initial stage of inflammation. Hence, chronic inflammation and infections in undernourished children may exert prolonged pressure on systemic tryptophan levels. Coupled with the enhanced consumption of tryptophan by the gut microbiome of these individuals this may have detrimental consequences for the regulation of growth, behavior, cognition and immune responses.

The principal branch of the kynurenine pathway generates quinolinic acid and nicotinamide. In humans, nicotinamide is methylated by the SAMe-dependent enzyme, nicotinamide *N*-methyltransferase (NNMT), to form *N*-methylnicotinamide (NMND), which is further metabolized to 2-PY. Recently, NNMT has been identified as a novel regulator of energy expenditure where knockdown of NNMT expression increased nicotinamide and SAMe^37^. Elevations in nicotinamide, a NAD^+^ precursor, have been shown to increase energy expenditure and by entering the polyamine flux, SAMe has also been shown to have a similar metabolic effect^38^. By increasing energy expenditure NNMT knockdown protected against diet-induced obesity. Here, undernourished children excreted greater amounts of 2-PY than the control children. This may reflect a metabolic adaptation in these children to increase NNMT activity and reduce nicotinamide and SAMe abundance, thereby lowering energy expenditure. Intriguingly, stunted children or those at risk of stunting who excreted greater amounts of NMND and 2-PY at baseline were found to have better growth 2 to 5 months later. These children also excreted lower amounts of β-aminoisobutyric acid at baseline, another metabolite involved in the regulation of energy expenditure. Through PPARα-mediated increases in white adipocyte thermogenesis and increased hepatic fatty acid β-oxidation, β-aminoisobutyric acid has been shown to increase energy expenditure and protect against diet-induced obesity^39^,^40^. Similarly, children with better growth excreted lower amounts of this metabolite. As with the changes observed in NMND and 2-PY excretion, this metabolic alteration could potentially reflect a reduction in energy expenditure. Hence, the better growers may adapt more effectively than those with poorer growth to their nutritionally limited environment by reducing energy expenditure in favor of growth. This metabolic adaption could predispose the individual to higher risk of obesity or insulin resistance in later life. Indeed, up-regulation of NNMT activity and a subsequent increase in plasma NMND has been observed with atherosclerosis and insulin resistance and increases in urinary NMND and 2-PY have been reported with type 2 diabetes^15^,^41^,^42^. Up-regulation of NNMT in cancer cells has also been found to impair SAMe-mediated methylation of DNA and histones^43^. As such, increased NNMT activity in these children could place additional pressure on their methylation capacity with epigenetic consequences.

Using a metabolic phenotyping approach we have shown disruptions to choline and tryptophan metabolism that could explain later-life outcomes of early-life undernutrition and potential effects on the epigenome due to the reduced methylation capacity of undernourished children. Furthermore, metabolic adaptation may occur in stunted children to restrict energy expenditure to promote growth with potential implications for later life. As markers of this adaptation, urinary NMND and β-aminoisobutyric acid may provide useful non-invasive biomarkers for growth propensity in children at risk of stunting.

## Methods

### Population and study design

The children participating in this study were mainly from Fortaleza and neighbouring municipalities in Ceará, Brazil. The study was conducted from August 2010 to September 2013 at the Institute for the Promotion of Nutrition and Human Development (IPREDE) in collaboration with the Institute of Biomedicine, Faculty of Medicine, Federal University of Ceará. This case-control study was part of the Malnutrition-Enteric Diseases (MAL-ED) network and the protocol and consent form were approved by the local institutional review board at the Federal Universisty of Ceará, the national IRB Conselho Nacional de Ética em Pesquisa (CONEP), and the University of Virginia in the United States. All work was done in accordance with these relevant guidelines and regulations and informed consent was obtained from the responsible guardians of all subjects.

Children who were being seen in the IPREDE clinic whose screening WAZ was <−2 were invited to participate, consent (parental or guardian) and to return to enrol. Approximate (i.e. within 6 months) age and gender matched ‘control’ children whose WAZ was >−1 were then invited to participate, consent and return to enrol. The inclusion criteria were as follow: (a) 6–24 months of age; (b) cases defined as CDC/WHO weight-for-age *z* score <−2 and controls >−1; (c) being healthy (*i.e.,* without any specific illness or fever); (d) mother/primary care-giver present and have legal custody of the child. The exclusion criteria were as follows: (a) children with serious health issues or who require prolonged hospitalization; or (b) a parent or care-giver with cognitive deficits or who was <16 year old. Children were enrolled after the parents or guardians of children who met the entry criteria provided informed consent. Cases and controls had their height and weight measured at enrolment and, when possible, at follow up 2–5 months later at the IPREDE clinic. An initial power analysis was used to estimate that 200 each of Cases and Controls should be sufficient to detect expected differences between undernourished and nourished children. The same number of Cases (201) and Controls (201) as well as equal numbers of boys and girls were enrolled. At their first visit, urine was collected and a 4-hour lactulose-mannitol urinary excretion test of gut function was performed. Of the 402 children enrolled, 337 provided adequate urine samples for metabolic analyses.

### ^1^H NMR spectroscopy

All urine samples were analyzed by ^1^H nuclear magnetic resonance (NMR) spectroscopy. For spectroscopic analysis, 400 μL of urine was combined with 200 μL of phosphate buffer (pH 7.4; 100% D_2_O) containing 1 mM of 3-trimethylsilyl-1-[2,2,3,3-^2^H_4_] propionate (TSP) as an external standard and 2 mM sodium azide as a bacteriocide. Samples were mixed by vortex and spun (13000 *g*) for 10 minutes prior to transferring 550 μL to a 5 mm NMR tube. Quality control samples were prepared by combining aliquots of urine (10 μL) from randomly selected individuals. Specimens were randomized and interspersed with quality control aliquots (using a total of 10 aliquots) in order to assess data quality and variation over the analytical measurement period. Spectroscopic analysis was carried out on a 700 MHz Bruker NMR spectrometer equipped with a cryo-probe. Standard one-dimensional ^1^H NMR spectra of the urine samples were acquired [recycle delay (RD)−90°−t_1_−90°−t_m_−90°-acquire free induction decay]. The water signal was suppressed by irradiation during the RD of 2 s, with a mixing time (t_m_) of 10 μs. The acquisition time was set to 2.91 s and the 90° pulse length was 15.87 μs. For each sample, 8 dummy scans were followed by 128 scans and collected in 64K data points using a spectral width of 16 ppm. Two-dimensional ^1^H–^1^H correlation spectroscopy, ^1^H–^1^H total correlation spectroscopy, ^1^H–^13^C heteronuclear single quantum coherence and J-resolved NMR spectra were acquired to aid metabolite identification in addition to statistical total correlation spectroscopy^44^.

Spectra were manually phased, corrected for baseline distortions and referenced to the TSP signal at δ 0.00. ^1^H NMR spectra (δ 0.2–10.0) were digitized into consecutive integrated spectral regions (~20,000) of equal width (0.00055 ppm). The regions between δ 4.7–6.10 containing the residual water resonance and the urea peak were removed from all spectra in order to minimize the effect of baseline effects caused by imperfect water suppression. For each spectrum a recursive segment-wise peak alignment (RSPA) algorithm was applied to minimize chemical shift variation due to residual pH differences within samples^45^. Each spectrum was then normalized to unit area to account for variation in sample concentration.

### Multivariate data analysis

Multivariate modeling was performed in Matlab using in-house scripts. This included principal components analysis using pareto scaling and orthogonal projection to latent structures (OPLS) constructed using unit variance scaling. Principal components analysis was first applied to visualize the global variance of the data sets to reveal intrinsic similarities in the spectral profiles and to identify outliers. Here, 11 samples were identified as outliers and removed from further analyses with 326 metabolic profiles remaining (data not shown). OPLS models were constructed to identify features in the urinary spectra associated with undernutrition. These features were then assigned to metabolites using in-house databases and 2D NMR experiments. Here, ^1^H NMR spectroscopic profiles were used as the descriptor matrix (X) and continuous anthropometric measures (e.g. height-for-age *z*-score (HAZ)) were used individually as the response variable (Y). Orthogonal signal correction filters remove the variation in the descriptor matrix that is unrelated to the response variable and thus assist model interpretation and aid identification of metabolites associated with the response variable. Loading coefficient plots were generated by back-scaling transformation to display the covariance between the Y-response matrix and the signal intensity of the metabolites in the NMR data (X). Hence, the direction and magnitude of the signals relate to the covariation of the metabolites with the Y-response in the model. Colors projected onto the coefficient plot indicate the correlation coefficient (r^2^) between each metabolite and the Y-response variable, with red indicating strong significance and blue indicating weak significance. The predictive performance (Q^2^Y) of the model was calculated using a seven-fold cross validation approach, model validity was established by permutation testing (1000 permutations) and the results are given as *p* values. ΔHAZ was calculated using follow-up HAZ measurements collected two to five months after baseline and used as a measure of growth in the children. An OPLS model was built using the metabolic profiles from children with a HAZ <−1 at baseline as the X-descriptor matrix and ΔHAZ as the Y-response variable. This allowed metabolic associations with catch-up growth in these children to be identified. This analysis was repeated using children with a HAZ <−2.

## Additional Information

**How to cite this article**: Mayneris-Perxachs, J. *et al.* Urinary *N*-methylnicotinamide and β-aminoisobutyric acid predict catch-up growth in undernourished Brazilian children. *Sci. Rep.* **6**, 19780; doi: 10.1038/srep19780 (2016).

## Supplementary Material

Supplementary Information

## Figures and Tables

**Table 1:** Summary of the orthogonal projection to latent structures (OPLS) models returned for the various measures of nutritional status.

Model	*n*	R^2^X	R^2^Y	Q^2^Y	*P*
height-for-age *z* score	326	0.045	0.14	0.035	0.001
weight-for-age *z* score	326	0.039	0.18	0.039	0.001
weight-for-height *z* score	326	0.035	0.20	0.016	0.002
ΔHAZ	252	0.031	0.19	−0.021	—
ΔHAZ (HAZ_0_>−1)	79	0.027	0.37	−0.41	—
ΔHAZ (HAZ_0_<−1)	173	0.048	0.20	0.028	0.0040
ΔHAZ (HAZ_0_<−2)	108	0.044	0.28	0.030	0.0190

ΔHAZ, change in height-for-age *z*-score over two to five months. HAZ_0_, height-for-age *z*-score at baseline.
