# Supplementary material for: Urinary N-methylnicotinamide and β-aminoisobutyric acid predict catch-up growth in undernourished Brazilian children
Source: Sci Rep. 2016 Jan 27;6:19780. doi: 10.1038/srep19780 (PMC4728399; doi:10.1038/srep19780)
Supplement: Supplementary Information [file srep19780-s1.doc]

**Urinary *N*-methylnicotinamide and β-aminoisobutyric acid predict catch-up growth in undernourished Brazilian children**

Jordi Mayneris-Perxachs^1^, Aldo A.M. Lima^2^, Richard L. Guerrant^2,3^, Álvaro M. Leite^2,4^, Alessandra F. Moura^2,4^, Noélia L. Lima^2,4^, Alberto M. Soares^2^, Alexandre Havt^2^, Sean Moore^5^, Relana Pinkerton^3^, Jonathan R. Swann^1^*

**Supplementary Information**

**Table S1:** Demographic information for children by Case versus Control status. Case are defined by WAZ < -2 and Controls by WAZ > -1.

|  | **Controls**  **(n = 168)*** | **Cases**  **(n = 158)*** | ***p*** |
| --- | --- | --- | --- |
| ***Child*** |  |  |  |
| *Gender (n (%) male)* | 83 (49) | 78 (49) | 1.00 |
| Screening Age (mean ± SD) | 12.3 ± 5.3 | 14.8 ± 5.5 | <0.001 |
| Birth WAZ (mean ± SD)  (by caregiver’s report) | -0.52 ± 1.52  **(n=164)** | -1.70 ± 1.63  **(n=153)** | <0.001 |
| Breastfeeding (n (%) yes) | 102 (61) | 85 (54) | 0.219 |
| ***Caregiver*** |  |  |  |
| Age (years) | 26.3 ± 6.4 | 25.7± 6.6  **(n=156)** | 0.379 |
| Years Education | 7.9 ± 3.1 | 8.2 ± 2.9 | 0.274 |
| Domestics In House (n (%) yes) | 12 (7.2)  **(n=167)** | 5 (3.2)  **(n=156)** | 0.137 |

**Missing data noted by variable.*

**Table S2**: Descriptive statistics for children included in the OPLS models comparing HAZ, WAZ, WHZ, and ΔHAZ.

| Model | *N* | Mean | Std. Deviation | Std. Error | Range | Min | Max |
| --- | --- | --- | --- | --- | --- | --- | --- |
| HAZss | 326 | -1.7060 | 1.37222 | .07600 | 9.78 | -7.05 | 2.73 |
| WAZss | 326 | -1.2055 | 1.52797 | .08463 | 8.43 | -5.47 | 2.96 |
| WHZss | 326 | -0.4173 | 1.40163 | .07763 | 6.77 | -3.38 | 3.39 |
| ΔHAZ | 252 | 0.0745 | 0.44405 | .02797 | 2.31 | -.92 | 1.39 |

HAZss indicates HAZ at ‘study start’; similarly for WAZss and WHZss. ΔHAZ is the difference in HAZ from study start to 2-5 months later at follow-up when available.

**Table S3: Comparison of the spectral peak integrals for urinary metabolites identified to vary between Case and Control children. Cases are defined as WAZ < -2.**

|  | **Controls**  **(*n* = 168)** | **Case**  **(*n* = 158)** | ***P^1^*** | ***P^2^*** |
| --- | --- | --- | --- | --- |
| ***Anthropometry*** |  |  |  |  |
| HAZ (mean ± SD) | -0.80 ± 0.078 | -2.67 ± 0.08 | <0.001 | <0.001 |
| WAZ (mean ± SD) | 0.04 ± 0.08 | -2.52 ± 0.05 | <0.001 | <0.001 |
| WHZ (mean ± SD) | 0.61 ± 0.08 | -1.51 ± 0.07 | <0.001 | <0.001 |
| ***Metabolites^3^*** |  |  |  |  |
| Betaine | 48.44 ± 0.96 | 45.47 ± 0.92 | 0.026 | 0.009 |
| Dimethylglycine | 26.80 ± 1.22 | 22.81 ± 0.83 | 0.008 | 0.021 |
| *N*-methylnicotinic acid | 1.55 ± 0.6 | 2.02 ± 0.22 | 0.077 | 0.006 |
| *N-*methyl-2-pyridone-5-carboxamide | 3.65 ± 0.16 | 4.03 ± 0.17 | 0.099 | 0.076 |
| 3-indoxyl sulfate | 3.16 ± 0.17 | 3.89 ± 0.12 | 0.003 | 0.001 |
| 2-hydroxyisobutyrate | 11.23 ± 0.18 | 12.26 ± 0.21 | <0.001 | <0.001 |
| 4-cresyl-sulfate | 15.14 ± 0.84 | 19.39 ± 1.10 | 0.002 | 0.001 |
| Phenylacetylglutamine | 31.54 ± 1.66 | 39.46 ± 2.17 | 0.004 | 0.002 |
| Hippurate | 47.85 ± 3.29 | 53.81 ± 3.04 | 0.185 | 0.056 |
| Citrate | 71.30 ± 2.6 | 62.05 ± 2.48 | 0.013 | 0.009 |
| Alanine | 40.97 ± 0.94 | 37.30 ± 0.88 | 0.005 | 0.002 |
| Creatine | 170.15 ± 5.94 | 200.18 ± 6.71 | 0.001 | 0.003 |
| Creatinine | 228.38 ± 2.27 | 222.27 ± 2.45 | 0.068 | 0.053 |
| Methylguanidine | 19.52 ± 0.20 | 19.21 ± 0.21 | 0.277 | 0.217 |
| *N*-acetylglycoprotein | 58.79 ± 0.86 | 55.18 ± 0.79 | 0.002 | 0.004 |
| Trimethylamine | 16.16 ± 0.36 | 15.14 ± 0.33 | 0.038 | 0.032 |

^1^ Independent samples t-test

^2^ Mann-Whitney U test

^3^ Arbitrary units (peak integral)

**
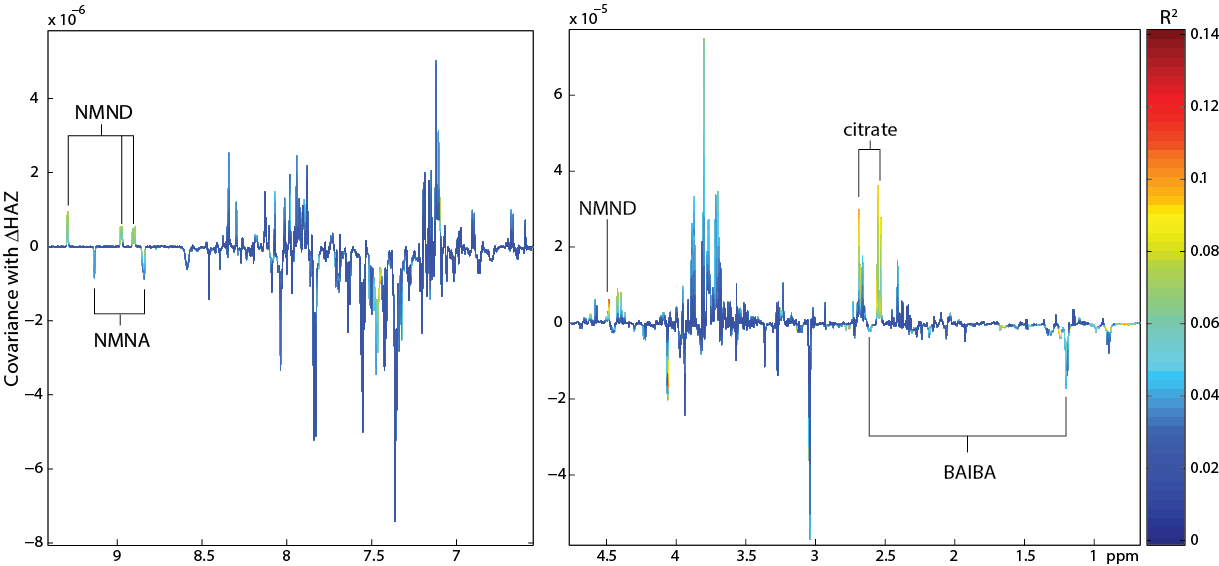
**

**Supplementary Figure S1:** OPLS coefficients plot identifying metabolic predictors of growth in stunted children. Baseline urinary metabolic profiles of children with a baseline HAZ < -2 were correlated with ΔHAZ measured two to five months later. BAIBA, β-aminoisobutyric acid; NMNA, *N*-methylnicotinic acid; NMND, *N*-methylnicotinamide.

**
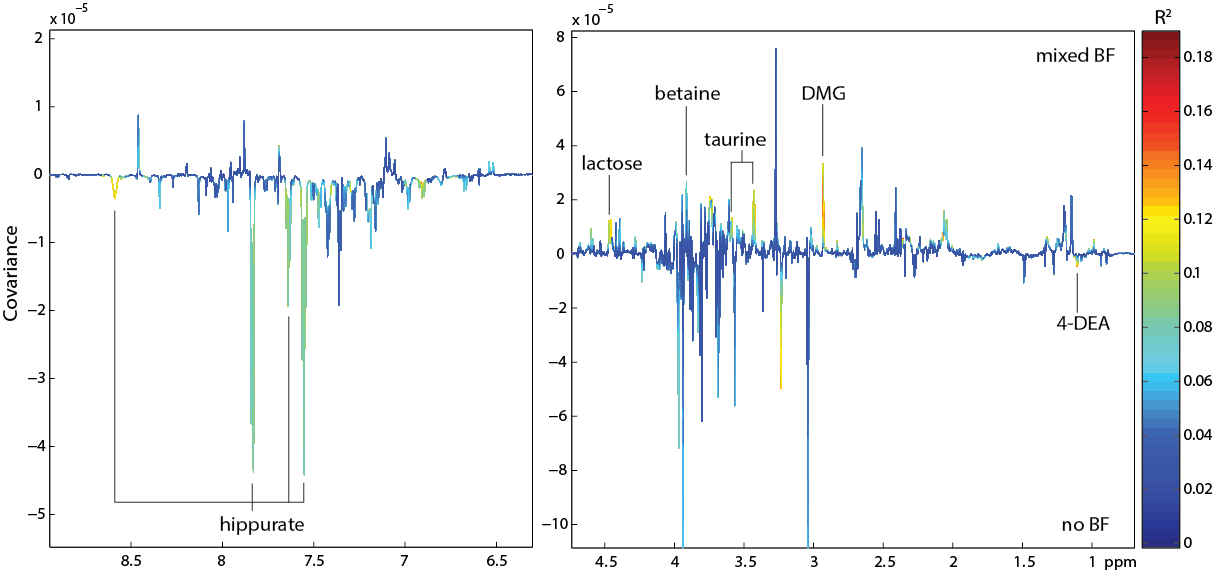
**

**Supplementary Figure S2:** OPLS-DA coefficients plot comparing the urinary metabolic profiles of children exposed to mixed breast-feeding practices versus those receiving no breast milk. DMG, dimethylglycine; 4-DEA, 4-deoxyerythronic acid.
